# Pharmacodynamic evaluation of intragastric pH and implications for famotidine dosing in the prophylaxis of non-steroidal anti-inflammatory drug induced gastropathy—a proof of concept analysis

**DOI:** 10.3109/21556660.2014.895371

**Published:** 2014-02-17

**Authors:** Jeffery D. Kent, Robert J. Holt, Donald Jung, George F. Tidmarsh, Amy Y. Grahn, Julie Ball, David A. Peura

**Affiliations:** 1 Horizon Pharma, Inc, Deerfield, ILUSA; 2 University of Illinois-Chicago, ILUSA; 3 Pharmaceutical Research Services, Inc, Cupertino, CAUSA; 4 Stanford University School of Medicine, Stanford, CAUSA; 5 University of Virginia Health Sciences Center, Charlottesville, VAUSA

**Keywords:** Famotidine, pH, Intragastric, NSAID, Gastropathy, Prophylaxis

## Abstract

**Objective:**

Famotidine given at a dose of 80 mg/day is effective in preventing NSAID-induced gastropathy. The aim of this proof of concept study was to compare twice a day (BID) vs 3-times a day (TID) administration of this total dose of famotidine on intragastric pH in healthy volunteers.

**Research design and methods:**

Two analyses were undertaken: (1) a 13 subject controlled cross-over 24-h intragastric pH evaluation of the BID and TID administration of 80 mg/day of famotidine, as well as measures for drug accumulation over 5 days (EudraCT, number 2006-002930-39); and (2) a pharmacokinetic (PK)/pharmacodynamic (PD) model which predicted steady-state famotidine plasma concentrations and pH of the two regimens.

**Results:**

For the cross-over study, gastric pH was above 3.5 for a mean of 20 min longer for TID dosing compared to BID dosing on Day 1. On Day 5, the mean time above this threshold was higher with the BID regimen by ∼25 min. For pH 4, subjects’ gastric pH was above this pH value for a mean of 25 min longer for TID dosing compared to BID dosing on Day 1. For Day 5, the pH was above 4 for ∼45 min longer with the TID regimen as compared with the BID regimen. The mean 24-h gastric pH values when taken in the upright position trended higher for the TID dosing period compared to the BID regimen on Day 1. The steady-state simulation model indicated that, following TID dosing, intragastric pH will be above 3 for 24 h vs 16 h for the BID regimen. There was no evidence for plasma accumulation of famotidine with TID dosing as compared to BID dosing from either analysis.

**Conclusion:**

The data indicate that overall more time is spent above the acidic threshold pH values when 80 mg/day of famotidine is administered TID vs BID. Key limitations included small study size with a short duration and lack of a baseline examination, but was compensated for by the cross-over and PK/PD modeling design. Although most of the comparisons in this proof of concept study were not statistically significant these results have important implications for future research on gastric acid lowering agents used for the prevention of NSAID-induced gastropathy.

## Introduction

Up to 50% of non-steroidal anti-inflammatory drug (NSAID) users demonstrate some degree of endoscopic gastrointestinal (GI) damage^1^. Endoscopic ulcers are considered early markers of more serious GI disorders such as perforation, obstruction, and bleeding, which may result in substantial morbidity and hospitalization^2^. Variability of NSAIDs in their inherent ability to cause GI ulceration has been reported both in controlled and observational trials^3,4^. Compared with non-use, all NSAIDs including the cyclooxygenase-2 inhibitor celecoxib have been associated with adverse upper GI events^4^. Furthermore, nuisance or minor GI side-effects, including nausea, dyspepsia, abdominal pain, flatulence, constipation, and diarrhea are common and affect 28–37% of patients who use NSAIDs to treat arthritic conditions^5,6^. Clinical trial data indicate that patients, such as those with osteoarthritis (OA) or rheumatoid arthritis (RA), administered high dosages of NSAIDs are 3-times more likely to experience dyspepsia^7^ and report discontinuation rates as high as 17%^8^.

Prevention of NSAID-induced GI damage and discontinuation due to GI adverse effects have been well documented with the concomitant administration of gastric acid inhibitors such as proton pump inhibitors (PPIs), histamine H_2_-receptor antagonists (H2RAs), and the GI cytoprotectant misoprostol^9–11^. Each of these agents has its unique mechanisms of action, adverse effects and recommended administration guidelines due to metabolic, pharmacodynamic, and potential drug interaction differences.

PPIs are potent gastric acid inhibitors and may be associated with unique long-term side-effects in some patients^12^. H2RAs may have to be given more frequently than PPIs due to shorter pharmacologic half-lives and potency, while misoprostol has significant gastrointestinal tolerability issues in some patients. Therefore, individual acid suppressants may be better suited for pairing with specific NSAIDs based on pharmacodynamic compatibility, individual patient needs and to improve compliance and adherence. For example, PPIs, such as esomeprazole, are once or twice a day (BID) drugs and are better paired with NSAIDs such as naproxen that are given once or twice a day and have a higher GI risk profile. H2RAs, such as famotidine, are more optimally paired with NSAIDs that are given 3-times a day (TID), such as ibuprofen. Misoprostol and diclofenac can be administered BID and up to 4-times a day (QID). Hence, three fixed dose combination products are currently approved in the US for the prevention of NSAID induced gastropathy.

For gastric acid suppressing drugs, previous research indicates that an intragastric pH of >3.5 is effective in preventing stress-induced ulcers and a pH >4 has been recommended for NSAID prophylaxis based on a murine model with indomethacin^13,14^. Furthermore, a pH >3 for 18–20 h/day for 3–4 weeks has been shown to be the optimal range of acid suppression for healing and prevention of recurrence of duodenal ulcers^15^. Controlled clinical trials have indicated that full daily doses of PPIs and double doses of famotidine (80 mg/day given TID or BID) are effective in significantly reducing the risk of NSAID-induced gastric ulcers, with significance established in 6–8 weeks^16–18^.

Since there are limited data evaluating famotidine in the context of the various pH thresholds, we conducted two analyses to specifically examine the pH effects of a known effective daily dose of famotidine (80 mg/day) when administered BID or TID to determine the intragastric pH effects of two daily dosing schedules of 80 mg/day of famotidine in preventing NSAID-induced gastropathy. The first was a randomized, open-label, two-period, cross-over, 5-day study in 13 healthy subjects and the second a pharmacokinetic (PK)/pharmacodynamic (PD) model based on previously conducted bioequivalence studies with famotidine alone and a fixed dose ibuprofen/famotidine combination. In the first study, the effects of an 80-mg daily dose of orally administered famotidine on intragastric pH were compared over 5 days between BID and TID dosing regimens. The study also evaluated differences in the trough plasma famotidine concentrations to determine if accumulation was occurring with the TID regimen. In the PK/PD analysis, data from two bioequivalence studies were used to predict mean plasma famotidine concentration vs time data of the commercial ibuprofen/famotidine combination tablet (administered TID) and famotidine alone (administered BID). Furthermore, intragastric pH effects of the two famotidine dosing schedules were modeled based on previously published plasma levels and degree of intragastric pH lowering. Therefore, these analyses were undertaken to examine whether a known clinically effective dose of famotidine (80 mg/day) for the prevention of NSAID associated gastropathy produced differing pH effects when given BID or TID.

## Patients and methods

### Gastric pH and the safety of 80 mg doses of famotidine administered BID vs TID

This Phase 1, single center, randomized, open-label, two-period, cross-over study was designed to compare the effects on gastric pH and the safety of 80 mg doses of famotidine (Commercial Famotidine Oral Suspension 40 mg/5 mL) when administered in two vs three divided doses each day. Thirteen healthy subjects (nine male, four female), average age 27.2 years (range = 21–41 years), were randomized to treatment. Subjects were assigned randomly, in approximately a 1:1 ratio, to one of two, two-period treatment sequences as follows: Treatment Sequence 1 (*n* = 6): 40 mg famotidine BID (5 mL BID), followed by 26.6 mg famotidine TID (3.33 mL TID). Treatment Sequence 2 (*n* = 7): 26.6 mg famotidine TID (3.33 mL TID), followed by 40 mg famotidine BID (5 mL BID).

There was a washout of at least 1 week between administration of the last dose of Treatment Period 1 and administration of the first dose of Treatment Period 2. All doses of study medication were administered orally, on an open-label basis. Famotidine Oral Suspension 40 mg/5 mL was purchased commercially and dispensed to study subjects from its original container. Famotidine TID was given at approximate hours 08:00, 16:00, and 24:00 on each day and BID was given at approximate hours 08:00 and 20:00. Subjects were prohibited from taking any medications or interventions that could decrease gastric acid secretion or neutralize gastric acid, and any medications that are known or suspected to cause dyspepsia or gastrointestinal ulcers, throughout the study period.

Subjects were screened within the 20 days prior to study entry and remained at the study center (Quintiles Uppsala AB, Sweden) beginning at ∼15:00 hour on Study Day 0 and continuing until ∼10:00 hour on Study Day 6 of both treatment periods. Subjects were followed for 14 days after administration of their last dose of study medication. Gastric pH was measured continuously using a nasogastric pH probe, during the 24 hours following administration of the first dose of study medication on Study Day 1 and during the 24 hours following administration of the first dose of study medication on Study Day 5 in both treatment periods^19^. Safety was assessed via adverse event reporting, physical examinations, and clinical laboratory assessments for all patients for 20 days. In order to assess any famotidine accumulation with the TID dosing schedule, trough concentration blood samples were collected prior to dosing on Day 1 and Day 5 of both treatment periods. Given that this was a proof of concept study, it was not expected to generate statistically significant results, however, one-sample, two proportion, double-sided T-tests, with a 95% confidence interval (CI), were performed on the BID and TID regimens for mean famotidine trough plasma concentrations and the time above the pH threshold values of 3.5 and 4.0 to determine trends. Mean pH values were used as the primary outcome based on previous H2RA research which indicates that it has high discriminating value^13^.

### Pharmacokinetic/pharmacodynamic and pH analysis of 80 mg/day of famotidine from a famotidine/ibuprofen combination administered TID compared with famotidine alone administered BID

In an analysis to further investigate the effects of famotidine administered BID or TID on steady-state intragastric pH, pharmacokinetic as well as pharmacodynamic modeling was performed using WinNonlin (version 5.3, Pharsight, Mountain View, CA). The mean plasma famotidine concentration vs time data following single dose oral administration of the commercial fixed dose combination ibuprofen 800 mg/26.6 mg famotidine formulation from Horizon Study HZ-CA-017 (IND #72116, Horizon Pharma, Inc., Deerfield, IL) and famotidine 40 mg (Pepcid, Merck & Co, Whitehouse, NJ) from a bioequivalence study (Teva Pharmaceuticals, ANDA 75311) were used for pharmacokinetic and pharmacodynamic assessments. A one compartment body model with first-order absorption/elimination and a lag time was fit to each set of mean observed data. A sigmoidal *E*_max_ model with a baseline effect parameter was used to fit the observed plasma famotidine concentration following an intravenous infusion of 0.1 mg/kg famotidine over 5 min vs intragastric pH data from that reported in the literature^20^.

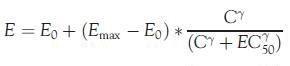

where *E* is intragastric pH, *E*_0_ is the intragastric pH at time zero (baseline pH), *E*_max_ is the maximal intragastric pH, *C* is the plasma famotidine concentration, *EC*_50_ is the plasma famotidine concentration at one-half *E*_max_ and *γ* is a slope factor.

Simulations of steady-state famotidine plasma concentration-time profiles were performed using the one compartment pharmacokinetic model described above following administration of ibuprofen/famotidine tablet formulation administered every 8 h (TID) and Pepcid every 12 h (BID)^21^. The simulated plasma famotidine concentrations together with the pharmacodynamics parameters obtained from the sigmoidal *E*_max_ model were then used to simulate the intragastric pH concentration vs time curve.

This model produced predicted famotidine plasma concentration-time profiles and intragastric-pH time profiles following oral administration of 80 mg/day of famotidine administered alone BID or TID in a fixed NSAID/famotidine combination. Area under the time curves (AUC) were calculated for the two famotidine treatment regimens and compared.

The intragastric pH study was approved by the Regional Ethics Committee of Uppsala, Sweden and registered with EudraCT, number 2006-002930-39. The bioequivalence study (HZ-CA-017) was approved by the St. Charles Institutional Review Board (St. Charles, MO). Both of these studies were conducted in compliance with the Declaration of Helsinki and its amendments, and the principles of Good Clinical Practice, including archiving of essential study documents.

## Results

### Cross-over intragastric pH study

**Figure 1.**
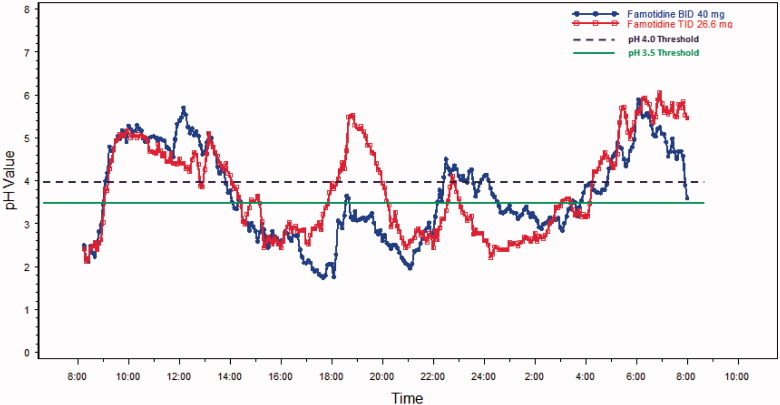
Mean pH values on Day 1.

**Figure 2.**
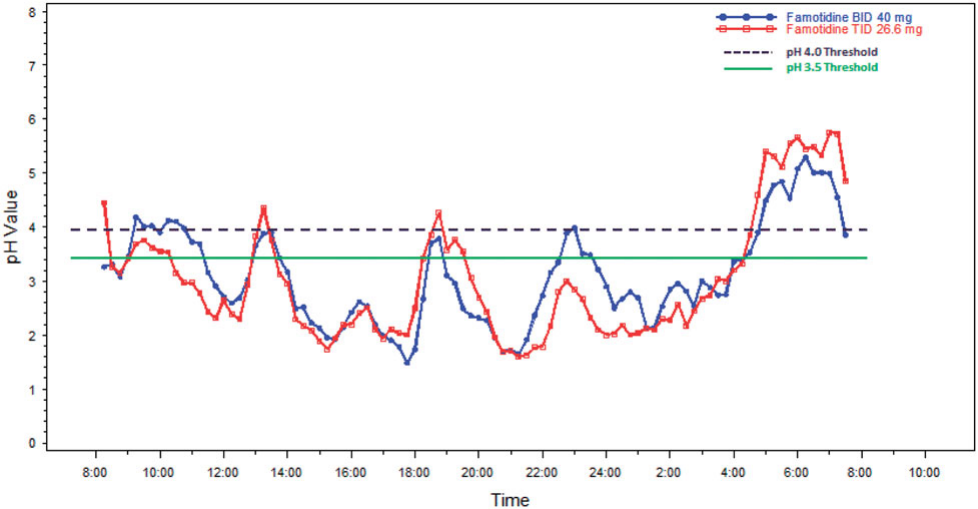
Mean pH values on Day 5.

The mean 24-h pH results over time are shown in Figures 1 and 2 for Day 1 and Day 5, respectively. On Day 1, for each threshold, subjects on average spent a shorter time in the acidic range during TID dosing compared to BID dosing of famotidine 80 mg/day. Mean time above the pH threshold of 3.5 was ∼20 min longer for the TID group, as compared to the BID group, and occurred predominately in the early evening just before the next BID dose was scheduled on the first day (see Figure 1) (*p* = 0.67, CI = −63, 102). On Day 5, the mean time above this threshold was higher, with the BID regimen by ∼25 min (See Figure 2, *p* = 0.60, CI = −128, 38). On Day 1, time above pH 4 was ∼22 min longer with the TID regimen as compared with the BID regimen (Figure 1, *p* = 0.58, CI = −54, 98). For Day 5, the pH was above 4 for ∼45 min longer with the TID regimen as compared with the BID regimen (see Figure 2, *p* = 0.31, CI = −30, 125).

The standard deviation, average absolute deviation and range were all smaller for TID dosing compared to BID dosing. The range of mean 24-h pH values for BID dosing was 1.8–5.1 pH units, or a 3.3 pH unit difference between the minimum value and the maximum value. By comparison, the range was 2.5–4.5 units or a 2.0 pH unit range, for TID dosing. This 1.3 pH unit range difference in the two dosing regimens trended in favor of the TID dosing regimen (*p*-value = 0.078).

There was no evidence for plasma accumulation of famotidine with TID dosing as compared to BID dosing on Day 5 (*p* = 0.99) (Table 1). Five subjects experienced a mild adverse event in either treatment schedule, while three patients experienced a moderate event (two with TID, one with BID regimen). The most common complaints were mild gastrointestinal events. There were no serious events reported.

**Table 1.** Trough famotidine concentrations.

	Trough plasma concentration of famotidine (ng/mL)			
	40 mg BID		26.6 mg TID	
	Day 1	Day 5	Day 1	Day 5
Mean	10.5	15.7	9.7	15.7
SD	2.8	4.6	4.9	8.9

### Steady-state PK/PD model

**Figure 3.**
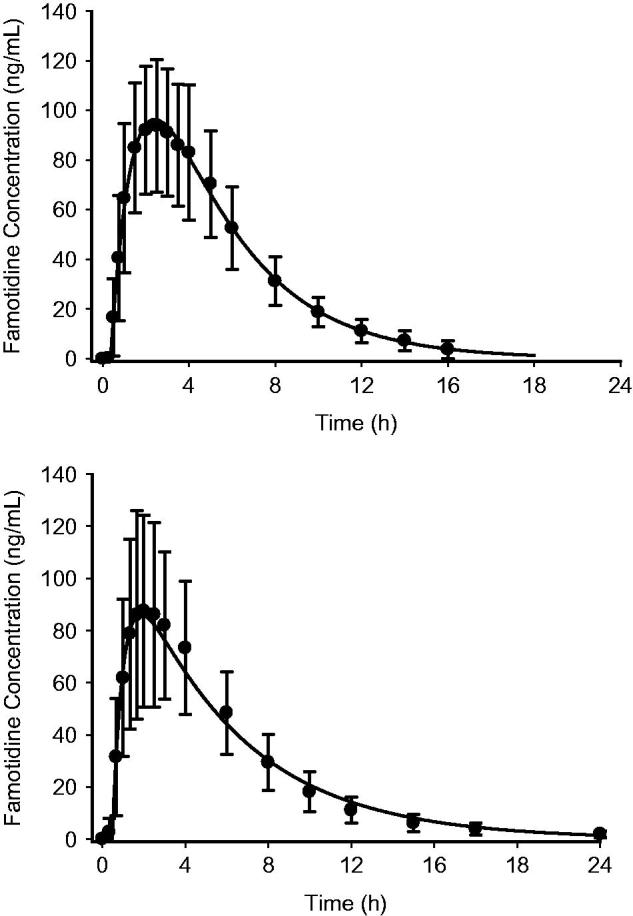
Mean observed vs fitted plasma famotidine concentrations single oral doses of 40 mg famotidine (Pepcid) (*n* = 30) (top) and ibuprofen 800 mg/famotidine 26.6 mg (*n* = 35) (bottom).

**Figure 4.**
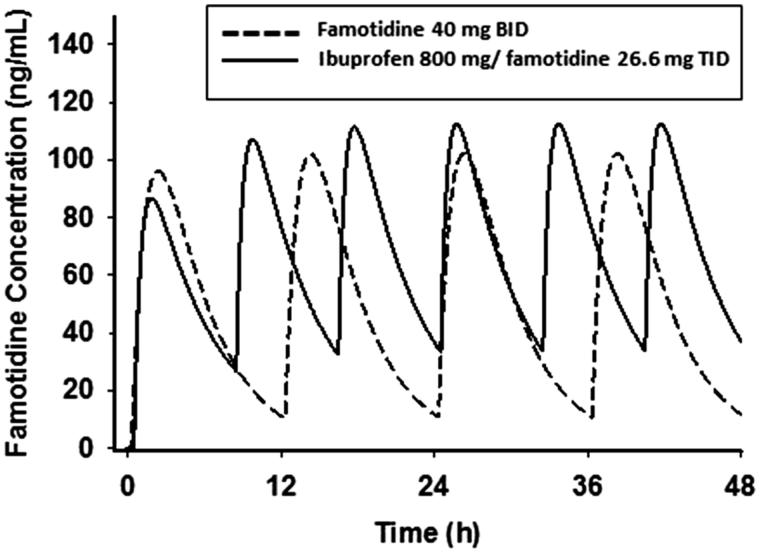
Predicted plasma concentration-time profiles of famotidine.

The mean plasma famotidine concentration vs time data following single dose oral administration of the fixed dose combination ibuprofen 800 mg/26.6 mg famotidine formulation and famotidine 40 mg (Pepcid) were fit to a one-compartment body model with first-order absorption/elimination and a lag time. The following pharmacokinetic parameters obtained from the non-linear least squares fit of the data (Table 2) were used to simulate the plasma famotidine concentrations following the two dosing regimens (Figure 3), where *V*/*F* is the apparent volume of distribution, *k_a_* is the absorption rate constant, *k_e_* is the elimination rate constant, and *t*_lag_ is the lag time. The predicted famotidine steady-state plasma concentration time profiles following the BID dosing of 40 mg famotidine and ibuprofen/famotidine 26.6 mg administered TID are shown in Figure 4. As expected, famotidine concentrations show greater peak-to-trough variations following famotidine 40 mg BID as compared to the ibuprofen/famotidine 26.6 mg combination given TID. The famotidine AUCs were 1249 and 1737 ng-h/mL, respectively, for famotidine 40 mg BID and ibuprofen/famotidine 26.6 mg TID.

**Table 2.** Pharmacokinetic parameters of different famotidine formulations.

Parameter	Famotidine^a^	Ibuprofen/famotidine combination^b^
*V*/*F* (L)	240	234
*k_a_* (h^−1^)	0.795	1.81
*k_e_* (h^−1^)	0.267	0.195
*t*_lag_ (h)	0.344	0.504

^a^40 mg famotidine, p.o. ^b^800 mg Ibuprofen/26.6 mg famotidine, p.o.

**Figure 5.**
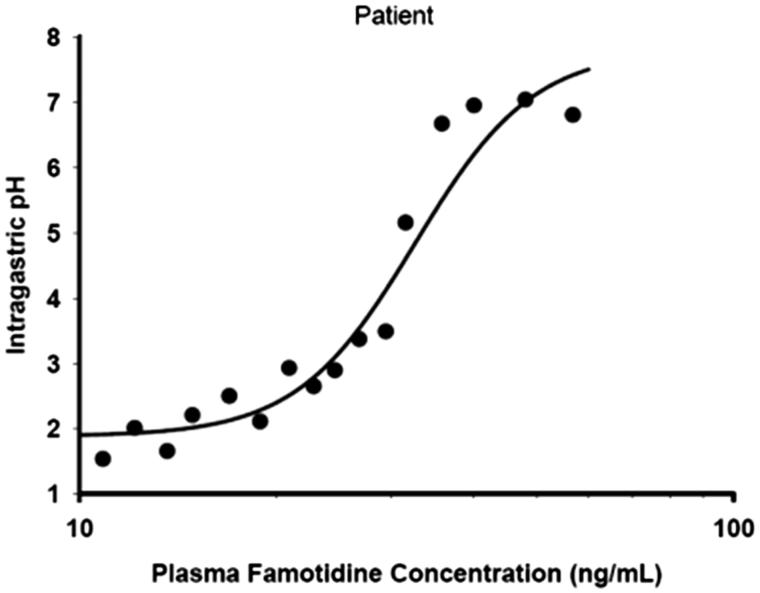
Predicted intragastric pH-time profiles of famotidine following a 5-min intravenous infusion of 0.5 mg/min famotidine.

The pharmacodynamic parameters obtained from the fit of the observed plasma famotidine concentration following an intravenous infusion of 0.1 mg/kg famotidine over 5 min vs intragastric pH data (Figure 5) were as shown in Table 3.

**Table 3.** Pharmacodynamic parameters for famotidine.

Parameter	
*E*_max_	7.11
*EC*_50_ (ng/mL)	30.60
*E*_0_	2.13
*Γ*	9.88

**Figure 6.**
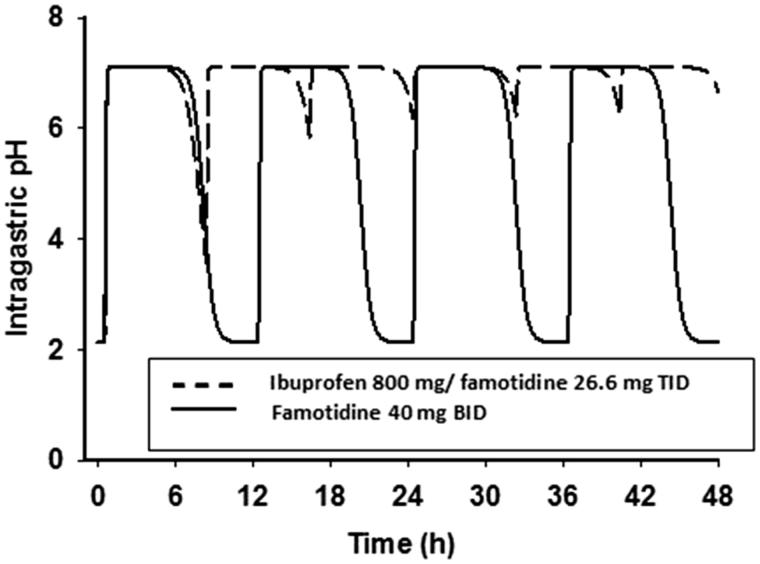
Predicted intragastric pH-time profiles of famotidine.

The predicted intragastric pH as a function of time is shown in Figure 6. As a result of the more frequent dosing with the ibuprofen/famotidine combination as compared to famotidine 40 mg BID, there is less fluctuation in intragastric pH during both a dosing interval and a 24-h steady-state period. Following BID dosing, intragastric pH will be above pH 3, 3.5, and 4 for 16.8, 16.4, and 16.0 h, respectively, while following TID dosing, intragastric pH will be above 3 for all 24 h. Famotidine concentrations greater than 26.2 ng/mL will keep pH above 3, while concentrations of greater than 27.7 and 29.1 will keep intragastric pH above 3.5 and 4, respectively. Based on differences in effective plasma concentrations for the two treatment regimens, a greater amount of time will be at lower intragastric pHs with the BID dosing regimen vs the TID regimen (Figures 4 and 6).

## Discussion

We report here the first analyses that suggest that a known effective daily dose of famotidine (80 mg/day) for the prevention of NSAID induced gastropathy produces differing intragastric pH effects when given TID vs BID to normal volunteers. Our data indicate that more time is spent above the acidic threshold pH values of 3.0, 3.5, and 4.0 with the TID than the BID regimen.

Previous research in GI healing studies have indicated that the mean duration of time with a pH >3 or pH increase >1 on the day of treatment with an H2RA have high statistical and clinical discriminating power^13^. This finding is supported by another study that found that a gastric pH 3 holding time ratio ≥∼50% might be required to prevent severe gastric mucosal injury induced by low dose aspirin^22^. In our population, this threshold was reached on both days 1 and 5 with both dosage regimens; however, the TID group had a higher percentage of mean time greater than pH 3 vs the BID group (Figures 1 and 2). Furthermore, our steady-state model predicted that the TID regimen would keep the pH above 3 throughout 24 hours vs ∼16 hours with the BID regimen. The clinical significance of this difference remains to be tested.

The importance of gastric pH in the genesis of indomethacin-induced gastric damage was demonstrated in a murine model where gastric pH was varied over a wide range of doses^14^. GI damage was substantially reduced when intragastic pH was maintained above 4^14,23^. Intragastric pH studies completed with esomeprazole when used as prophylaxis for naproxen induced GI damage show a dose-dependent increase in the percentage of time that gastric pH was above 4, ranging from 50–70% after 9 days of therapy^24^. During the first day of therapy, however, the mean percentage of time spent in this range of pH was low, with all doses 13–18%^24^. In our analysis, the TID famotidine regimen demonstrated a mean pH above 4 ∼ 50% of the time during Day 1 and according to our simulations would remain so during steady-state.

During the observation period, pH values were recorded during a variety of conditions such as sitting upright, lying asleep, during meals and just after a meal. Each of these conditions can impact the gastric pH in a different manner. Specifically, measurements taken while upright tend to be more consistent due to the position of the pH probe, while values taken during meals are influenced by the acidity of the food in the stomach. The Day 1 gastric pH values when taken in the upright position were ∼0.4 pH units higher for the TID dosing period compared to the BID dosing period for the entire population (3.38 vs 3.79), which constitutes a 4-fold difference in the amount of gastric acidity. This trended in favor of the TID regimen (*p* = 0.09).

The PK/PD model findings support the conclusion that higher levels of intragastric pH occur with the TID dosing schedule vs the BID dosing schedule. The intragastric pH AUCs were demonstrably higher with the TID dosing regimen (Figure 6). The famotidine plasma levels were predicted by the model to be lower with BID dosing as compared to TID dosing (Figure 4). As expected, as a result of the short half-life of famotidine, actual trough famotidine plasma levels taken in the cross-over study on Day 1 and Day 5 for our 13 healthy volunteers did not show any differences and, therefore, there was no evidence of drug accumulation with the TID vs the BID regimen.

As a proof of concept study, our analyses have some limitations. Firstly, our intragastric pH findings showed small non-significant differences in the pH thresholds of 3.5 and 4 between the BID and the TID regimens. This is not surprising as the clinical efficacy of both regimens has been established in the clinical literature^17,18^. However, higher pH values and higher percentages of time spent above pH 3 may lead to clinical differences when studied over a longer period of time^13,22^.

Secondly, our results are primarily driven by increases in pH during the night time (8 pm to 8am) with both treatment regimens. While it is known that famotidine produces more potent pH lowering when administered in the evening vs morning with single doses^13^, the placement of the pH probe may have influenced the results. In anticipation of this, we completed an analysis of the intragastric data in the upright position and it was in general agreement with the overall results, demonstrating a slightly higher pH with the TID regimen.

Thirdly, our study was small (*n* = 13) and included only healthy volunteers but it is within the range and scope of 24-hour intragastric studies reported in the literature with PPIs and H2RAs^13,20,22,24^. Furthermore, strengthening the design of the study each patient served as his own control in the cross-over design comparing the two dosing regimens. Additionally, we employed a pharmacodynamic model to see if our results would be concordant. PD models have well known limitations, but are useful for hypothesis generation and cross-reference validity for small studies such as that reported here. The results of our PK/PD model were supportive directionally to the cross-over study results and give us weighted confidence in our conclusion that 80 mg/day of famotidine given TID, as compared to BID, will produce at least equivalent, and possibly improved, pH control without drug accumulation.

Fourthly, we report slightly differing results from Day 1 to Day 5. While tachyphylaxis to H2RAs has been suggested, the mean pHs were not significantly different with either treatment regimen in this study. Furthermore, studies suggesting tachyphylaxis with famotidine have been at lower doses than that reported here^25^ and it is known that clinical studies with 80 mg/day of famotidine in the prevention of upper GI ulcers in NSAID users have demonstrated efficacy up to 24 weeks, suggesting durability of effect^17,18,26^.

## Conclusion

A known effective daily dose of famotidine (80 mg/day) for the prevention of NSAID induced gastropathy produces differing intragastric pH effects when given TID vs BID to normal volunteers. Our controlled study and pharmacodynamic model indicate that generally more time was spent above acidic threshold pH values of 3, 3.5, and 4.0 with the TID than the BID regimen. Although most of our comparisons in this proof of concept study were not deemed statistically significant, these data provide insight for future investigation and have potential implications on clinical practice and are supported by clinical studies assessing the prevention of gastrointestinal ulcers.

## Transparency

### Declaration of financial/other relationships

JK, AG, and JB are employees of Horizon Pharma, Inc. GT was an employee of Horizon Pharma, Inc at the time of this study. RH, DJ, and DP are currently, or have been, consultants for Horizon Pharma, Inc. JDA Peer Reviewers have no relevant financial relationships to disclose.

## Abbreviations

AUC: area under the curve
BID: twice a day
BMI: body mass index
CI: confidence interval
EudraCT: European Union Drug Regulating Authorities Clinical Trials
GI: gastrointestinal
H2RA: histamine H2-receptor antagonist
M: meter
NSAID: non-steroidal anti-inflammatory drug
OA: osteoarthritis
PD: pharmacodynamics
PK: pharmacokinetic
PPI: proton pump inhibitor
QID: 4-times a day
TID: 3-times a day
